# With a Little Help from ^31^P NMR: The Complete Picture on Localized and Long-Range Li^+^ Diffusion in Li_6_PS_5_I

**DOI:** 10.1021/acs.jpcc.1c06242

**Published:** 2021-10-11

**Authors:** Katharina Hogrefe, Isabel Hanghofer, H. Martin R. Wilkening

**Affiliations:** Institute of Chemistry and Technology of Materials, Christian Doppler Laboratory for Lithium Batteries, Graz University of Technology (NAWI Graz), Stremayrgasse 9, AT-8010 Graz, Austria

## Abstract

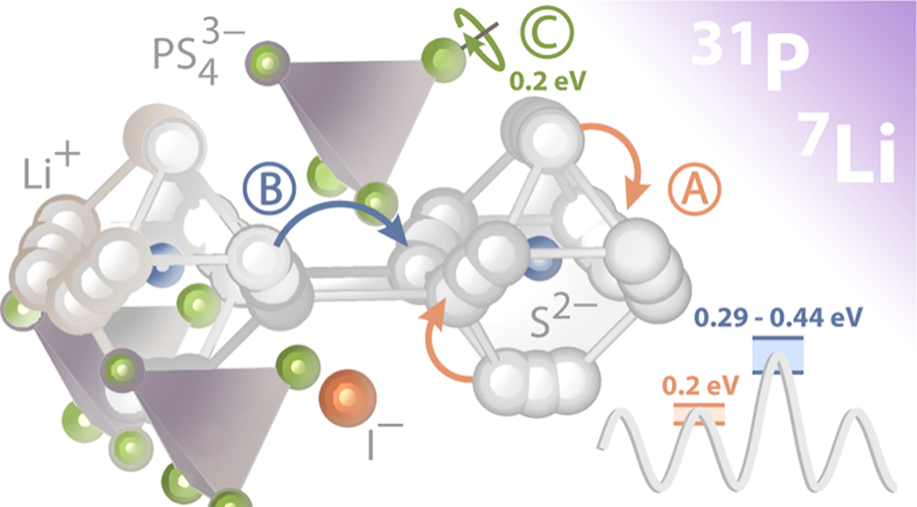

Li_6_PS_5_I acts as a perfect model substance to study length scale-dependent diffusion parameters in an ordered matrix. It provides Li-rich cages which offer rapid but localized Li^+^ translational jump processes. As jumps between these cages are assumed to be much less frequent, long-range ion transport is sluggish, resulting in ionic conductivities in the order of 10^–6^ S cm^–1^ at room temperature. In contrast, the site disordered analogues Li_6_PS_5_X (X = Br, Cl) are known as fast ion conductors because structural disorder facilities intercage dynamics. As yet, the two extremely distinct jump processes in Li_6_PS_5_I have not been visualized separately. Here, we used a combination of ^31^P and ^7^Li NMR relaxation measurements to probe this bimodal dynamic behavior, that is, ultrafast *intra*cage Li^+^ hopping and the much slower Li^+^*inter*cage exchange process. While the first is to be characterized by an activation energy of ca. 0.2 eV as directly measured by ^7^Li NMR, the latter is best observed by ^31^P NMR and follows the Arrhenius law determined by 0.44 eV. This activation energy perfectly agrees with that seen by direct current conductivity spectroscopy being sensitive to long-range ion transport for which the intercage jumps are the rate limiting step. Moreover, quantitative agreement in terms of diffusion coefficients is also observed. The solid-state diffusion coefficient *D*_σ_ obtained from conductivity spectroscopy agrees very well with that from ^31^P NMR (*D*_NMR_ ≈ 4.6 × 10^–15^ cm^2^ s^–1^). *D*_NMR_ was directly extracted from the pronounced diffusion-controlled ^31^P NMR spin-lock spin–lattice relaxation peak appearing at 366 K.

## 1 Introduction

The search for powerful solid electrolytes that can be used in sustainable Li-ion and Na-ion energy storage systems has reached an unpreceded level today.^1−5^ To identify and understand the origins behind fast ion transport in crystalline and amorphous solids, model substances are needed that allow the characterization of the distinct dynamic processes in detail, without any interfering effects from other diffusion processes taking place at the same time.

**Figure 1:**
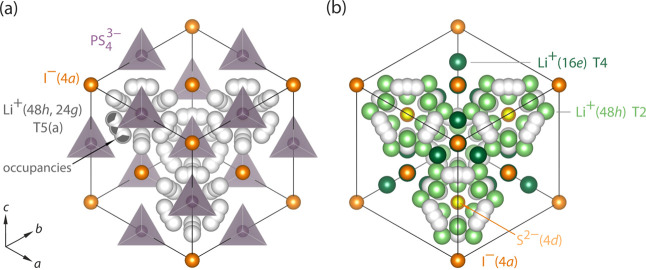
(a) Crystal structure of argyrodite-type, anion-ordered Li_6_PS_5_I. The I^–^ anions occupy the 4*a* sites, while the S^2–^ anions reside on the 4*d* and the 16*e* sites forming an ordered anion sub-lattice; for the sake of clarity, not all the S^2–^ anions are shown. Li^+^ ions are arranged such in the iodide compound that they build cages consisting of 48*h*-24*g*-48*h*′ triplets, also called T5a sites. These sites are only partially occupied by Li ions, as illustrated, enabling them to quickly jump within the cages, that is, within the triplets and between them. Jumps within the pocket-like triplets are to be considered as spatially highly confined. (b) Long-range Li^+^ ion dynamics in Li_6_PS_5_I is either possible via direct jumps between the cages or via interstitial sites. Interstitial sites are illustrated as green spheres [labelled T4 and T2 corresponding to the Wyckoff sites 16*e* (dark green) and 48*h* (light green)].

Li_6_PS_5_I belongs to the well-known group of argyrodite-type solid electrolytes^6−21^ that is assumed to host two dynamically distinct Li^+^ diffusion processes.^22,23^ In the anion-ordered Li_6_PS_5_I, whose structure is depicted in Figure 1, Li-rich cages are present that provide the opportunity of rapid localized Li^+^ hopping processes.^22,23^ Intercage jumps are, however, assumed to be much less frequent.^22,23^ This assumption was used to explain the poor long-range ion transport properties of Li_6_PS_5_I that shows ionic conductivities in the order of only 10^–6^ S cm^–1^ at room temperature.^22^ In contrast, site disorder, involving X^–^, S^2–^, and Li^+^, as is present in Li_6_PS_5_X (X = Br and Cl),^12^ switches on rapid intercage ion dynamics, turning these analogues into extremely fast ion conductors^6,11,14−17,19−24^ with specific conductivity values reaching 3.8 × 10^–3^ S cm^–1^ (Li_6_PS_5_Cl) and 2.2 × 10^–3^ S cm^–1^ (Li_6_PS_5_Br).^22^ As yet, the bimodal diffusion behavior suggested for Li_6_PS_5_I has, however, not been experimentally verified or even quantified with methods that are able to directly probe Li^+^ at the atomic scale. Here, we used a combination of ^7^Li and ^31^P nuclear magnetic resonance (NMR) spin–lattice relaxation techniques^24−28^ to conclusively characterize this unique dynamic property in Li_6_PS_5_I.

It turned out that, also based on earlier experiments in our group,^22^ the localized intracage dynamic process is to be characterized by an activation energy of only 0.2 eV. Jump rates at ambient conditions reach values in the order of 10^9^ s^–1^. This process is best seen by using ^7^Li NMR spectroscopy.^22,29^ Spin-lock ^31^P NMR relaxometry is, however, highly suitable to detect much slower Li^+^ diffusion process.^17,18,24^ Thereby, the ^31^P nuclei indirectly sense the ^7^Li spin fluctuations caused by the Li translational motions. Thus, we use an immobile nucleus from the framework to probe the irregular movement of the mobile Li^+^ ions, which is, for example, similar to that reported in the study of Kim et al.,^30^ who studied Li ion dynamics in borate glasses via both ^7^Li and ^11^B NMR relaxation measurements. Here, we were able to quantify the dynamic parameters from diffusion-induced ^31^P spin-lock NMR measurements and to compare them with results from direct current (dc) conductivity spectroscopy being sensitive to macroscopic, long-range ion transport.^22,31^ While classical laboratory-frame NMR spin–lattice relaxation measurements are sensitive to dynamic processes with 1/τ_NMR_ rates in the MHz to GHz range, spin-lock NMR is sensitive to atomic motions with jump rates 1/τ_NMR_ in the kHz range.^24,25,32^

In earlier studies of our group, we were able to identify two types of motional processes in Li_6_PS_5_I, viz., the fast Li^+^ intracage process and rotational dynamics of the polyanions. However, the origin of a shallow diffusion-induced peak in spin-lock ^7^Li NMR appearing at 360 K remained unclear. With the help of spin-lock ^31^P NMR, we are now able to explain this feature. The current study provides a full picture of Li^+^ ion diffusion in the high-temperature modification of Li_6_PS_5_I and reveals three different types of motional processes. Spin-lock ^31^P NMR is able to characterize the important intercage Li^+^ exchange process that is directly related to long-range ion transport in Li_6_PS_5_I. In general, the provision of such information is highly desirable if we want to understand length-scale dependent dynamic properties of even more complex materials that are currently being developed for their use as electrolytes in all-solid-state battery systems.

## 2 Methods

For the present study, we used a Li_6_PS_5_I sample of the same synthesis batch that has been investigated by our group recently.^22^ Thus, we refer to the literature^22,29^ for details on sample preparation and structural characterization.

The procedures to record variable-temperature ^7^Li (spin-quantum number *I* = 3/2) and ^31^P (*I* = 1/2) NMR (spin-lock) spin–lattice relaxation rates are identical to those described elsewhere by our group.^17,18,22,29^ We used a Bruker 300 MHz NMR spectrometer in combination with a Bruker broadband probe to record the laboratory-frame rates 1/*T*_1_ with the saturation recovery pulse sequence at magnetic fields *B*_0_ corresponding to Larmor frequencies ω_0_/2π in the MHz range. In this sequence, a train of 10 π/2 pulses destroys any longitudinal magnetization *M*_*z*_. The diffusion-induced recovery of *M*_*z*_ is immediately recorded as a function of waiting (or delay) time *t*_d_ and temperature *T* with a single π/2 detection pulse. Here, we analyzed the area under the free induction decays (FIDs) to construct *M*_*z*_(*t*_d_, *T*). The transients *M*_*z*_(*t*_d_) can be very well parameterized with stretched exponentials to extract the rate 1/*T*_1_ at Larmor frequencies of 116 MHz (^7^Li) and 121 MHz (^31^P). Here, the ^31^P NMR curves follow simple exponential behavior. Usually 4 to 16 scans (quadrature detection) were accumulated to obtain a single FID per waiting time; a complete curve *M*_*z*_(*t*_d_), containing 1/*T*_1_(*T*), consisted of 16 to 30 data points.

The corresponding NMR relaxation rates in the rotating (ρ) frame of reference 1/*T*_1ρ_ were acquired at a spin-lock frequency of 20 kHz by taking advantage of the two-pulse spin-lock technique.^18^ An initial π/2 pulse flips the longitudinal magnetization into the (*xy*) plane; immediately after that, the locking pulse with varying duration *t*_lock_ fixes the transversal magnetization *M*_(*xy*)′_(*t*_lock_).^18^ Spin relaxation in the presence of this weak magnetic field *B*_1_ (≪ *B*_0_) with values in the kHz range results in magnetization transients following a stretched exponential decay from which the diffusion-induced rate 1/*T*_1ρ_ was extracted. γ in *M*_(*xy*)′_ ∝ exp(−(*t*_lock_/*T*_1ρ_)^γ^) shows a mean value of 0.90(5) which does not indicate strong deviation from exponential behavior characterized by γ = 1. Details on this technique are given elsewhere.^17,18,25^

## 3 Results and Discussion

**Figure 2:**
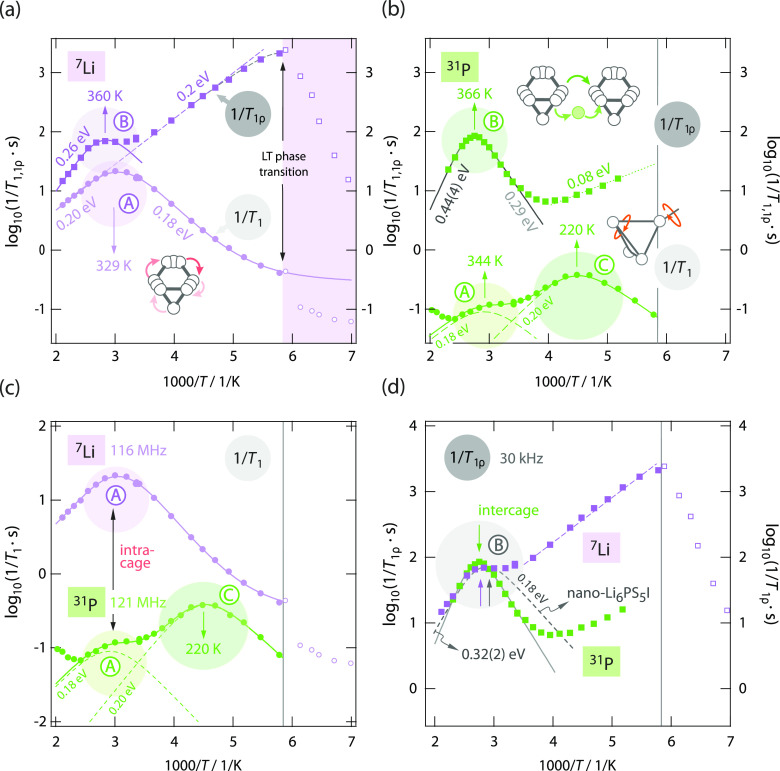
(a) ^7^Li NMR spin–lattice relaxation rates of Li_6_PS_5_I recorded at 116 MHz (1/*T*_1_) and 20 kHz (1/*T*_1ρ_), respectively. Solid lines use BPP-type spectral density functions to analyze the shape of the peak, taking into account a power-law behavior 1/*T*_1_ ∝ *T*^p^ at low temperatures. The dashed line represents a linear fit. (b) Corresponding ^31^P NMR spin–lattice relaxation rates of Li_6_PS_5_I recorded at 121 MHz (1/*T*_1_) and 20 kHz (1/*T*_1ρ_), respectively. Dashed lines refer to the individual 1/*T*_1_ peaks of the overall ^31^P 1/*T*_1_ NMR response which is approximated with a sum of two sub-peaks (solid line). The 1/*T*_1ρ_ response is approximated with a spectral density, taking into account correlated 3D motion (solid line). The dotted line represents a linear fit to approximate the behavior of the ^31^P 1/*T*_1ρ_ NMR rates at lower *T*. (c) Comparison of the ^7^Li and ^31^P NMR spin–lattice relaxation rates of Li_6_PS_5_I. (d) Arrhenius plot to compare the ^7^Li and ^31^P 1/*T*_1ρ_ NMR rates recorded in the rotating frame of reference. The dashed line indicates the diffusion-induced ^31^P NMR spin-lock rate peak of nanocrystalline Li_6_PS_5_I. Arrows shown and temperatures indicated refer to the positions of the individual peaks. Whereas the peaks labeled with A mirror fast *intra*cage Li^+^ dynamics [see (c)], the much slower *inter*cage ion dynamics are probed by the spin-lock peaks marked with B; see (d).

Figure 2 gives an overview of the ^7^Li and ^31^P (spin-lock) NMR spin–lattice rates of polycrystalline Li_6_PS_5_I. In Figure 2a, the ^7^Li NMR rates 1/*T*_1_ and 1/*T*_1ρ_ of Li_6_PS_5_I are shown using an Arrhenius representation. As discussed earlier,^22^ 1/*T*_1_ passes through a prominent diffusion-induced rate peak (labeled peak A) that is located at *T* ≈ 330 K. At this temperature, the mean jump rate 1/τ_NMR_ is in the order of the angular Larmor frequency ω_0_ (ω_0_τ_NMR_ ≈ 1),^25^ which is given by 2πν_0_ = 7.3 × 10^8^ s^–1^, thus reaching values in the GHz regime. Such fast exchange processes would result in ion conductivities in the mS range at 329 K. As this is not the case for Li_6_PS_5_I, the NMR peak was interpreted to mirror ultrafast but localized *intra*cage jump processes.^22^ These are thermally activated by 0.18–0.20 eV, as it can be deduced from analyzing the flanks of the 1/*T*_1_ NMR peak. The corresponding spin-lock NMR peak (A′) is expected to be seen at much lower temperatures. Indeed, below 200 K, the rates 1/*T*_1ρ_ reveal the high-temperature flank of this peak. Extrapolating this flank toward higher temperatures shows that it will coincide with the corresponding one of the 1/*T*_1_ peak (0.2 eV, see the dashed line in Figure 2a). The maximum of the ^7^Li spin-lock NMR 1/*T*_1ρ_(1/*T*) peak is expected at ca. 165 K, which is in agreement with the curvature of the dashed line indicated in Figure 2a. However, because of the phase transformation Li_6_PS_5_I is undergoing, an abrupt change of the ^7^Li NMR rates 1/*T*_1(ρ)_ is observed at temperatures lower than 170 K. Below 170 K, the 1/*T*_1ρ_ rates mirror the low-temperature flank (negative slope) of a diffusion-induced peak that reveals much lower Li^+^ diffusivity in the low-*T* modification of Li_6_PS_5_I.^9,10^

Most interestingly, the ^7^Li NMR rates 1/*T*_1ρ_ of Li_6_PS_5_I pass through a shallow maximum at 360 K (peak B), whose origin is still unclear.^22^ As we will show below, this peak reflects the rate-limiting Li^+^ hopping processes connecting the Li-rich cages enabling long-range ion transport in Li_6_PS_5_I.

Turning to ^31^P NMR spin–lattice relaxation (see Figure 2b), we recognize that the rate 1/*T*_1_ passes through two different maxima located at approximately 220 K (peak C) and 344 K (again labeled as peak A). As we have shown earlier,^29,33^ peak C, most likely, reflects rotational motions of the polyanions as it is unique to ^31^P and is not seen in ^7^Li NMR. On the other hand, the ^31^P NMR peak located at 330 K (peak A) mirrors the fast Li^+^ translational *intra*cage jumps that also dominate the 1/*T*_1_^7^Li NMR response (see peak A in Figure 2a).^29,33^ A comparison of the two peaks detected by ^7^Li and ^31^P NMR is given in Figure 2c. Note that the ^7^Li and ^31^P rates were recorded at almost the same Larmor frequencies (116 MHz vs 121 MHz), which is important for such a direct comparison, because to fulfil the condition ω_0_τ_NMR_ ≈ 1 (see above), the position of the peaks will shift with increasing ω_0_ toward higher temperatures. Here, the ^31^P nuclei are used as spies to indirectly sense the (^31^P–^7(6)^Li) spin fluctuations in their direct neighborhood, which are produced by Li^+^ translational motions. Importantly, the ^31^P magic angle spinning NMR spectrum^22^ shows only a single P-site for which the relaxation occurs.

It has to be mentioned that the two nuclei (^7^Li, *I* = 3/2; ^31^P, *I* = 1/2) are subjected to different interactions; whereas ^7^Li senses electric quadrupolar and (Li–Li, Li–P) magnetic dipolar interactions, ^31^P NMR relaxation is mainly influenced by the heteronuclear, dipolar (P–Li) interactions. Naturally, these differences result in distinct motional correlation functions seen by the NMR active spins. Estimating the effect on motional correlation times τ_c_ show that the differences in τ_c_ do not exceed a factor of five, which hardly affects the position of the corresponding rate peaks.

In the present case, ^31^P 1/*T*_1_ NMR is able to probe two different motional processes, *viz*., (i) the fast translational *intra*cage dynamics (seen by ^7^Li and ^31^P NMR) and (ii) the even faster rotational motions of the PS_4_^3–^ polyanions (seen by ^31^P NMR). The two corresponding peaks in spin-lock 1/*T*_1ρ_^31^P NMR measurements are expected to appear at much lower temperatures, that is, lower than 160 K to satisfy the condition ω_1_τ_NMR_ ≈ 1. Here, when coming from high temperatures, we do only see the beginning of a mutual high-temperature flank of these peaks that is activated by approximately 0.08 eV; see Figure 2b.

Most importantly, the spin-lock ^31^P NMR rates 1/*T*_1ρ_, measured in the rotating frame of reference (Figure 2b), reveal a prominent diffusion-induced peak at 360 K, which is exactly the temperature at which the shallow peak in spin-lock ^7^Li 1/*T*_1ρ_ NMR appears (360 K); a comparison is shown in Figure 2d. This ^31^P NMR peak reflects spin fluctuations pointing to much slower motional processes as those seen by 1/*T*_1_. The corresponding 1/*T*_1_^31^P NMR peak is expected to appear at a higher *T*; indeed, the ^31^P NMR rates 1/*T*_1_ reveal the beginning of a new low-*T* flank at temperatures higher than 415 K. In contrast to the ^7^Li 1/*T*_1ρ_ NMR peak that is seen at 360 K, the spin-lock ^31^P NMR rates are not affected by the laboratory-frame ^31^P 1/*T*_1_ NMR rates. The rates 1/*T*_1ρ_ and 1/*T*_1_ of the ^31^P spins differ by more than 2 orders of magnitude (Figure 2d), which is not the case for ^7^Li; see Figure 2a. Hence, the high-temperature flank of the shallow peak seen in 1/*T*_1ρ_^7^Li NMR underestimates the activation energy on the high-*T* side of this peak. This comparison reveals the advantages of using the ^31^P spin to indirectly sense the Li^+^ dynamics.

In the following, we will analyze the properties and origins of the spin-lock 1/*T*_1ρ_(1/*T*) ^31^P NMR peak in more detail. First of all, it turned out to be asymmetric in shape. The solid line shows a parameterization of the spin-lock NMR response with a spectral density function *J* commonly used to describe correlated 3D motion in solids.^28^*J*^3D^ relies on the well-known Lorentzian-shaped function *J*_BPP_ introduced by Bloembergen, Purcell, and Pound^34,35^ and is, in general, given by *J*^3D^(ω_0_, *T*) ∼ τ_c_/[1 + (ω_0(1)_τ_c_)^β^].^25^ For β = 2, this function is a special case of the more general Cole–Cole distribution function *J*_CC_ that also contains a width parameter δ to take into account a distribution of Debye spectral densities.^36^ For δ = 1, the Cole–Cole spectral density function *J*_CC_ yields *J*_BPP_ (= *J*^3D^) with β = 2. In general, the motional correlation rate 1/τ_c_ is expected to be identical with the jump rate 1/τ_NMR_ within a factor of two. The asymmetry parameter β describes the deviation from symmetric behavior of the peak.^28,37,38^ A symmetric peak is expected for uncorrelated 3D motion (β = 2) which produces a quadratic dependence of 1/*T*_1_ on the Larmor frequency ω_1_/2π on the low temperature side (ω_1_τ_c_ ≫ 1).^25,28^ Here, the peak is characterized by β = 1.65 (see Table 1). Such asymmetric peaks are described by a high-*T* (*E*_a, high_ ≡ *E*_a, NMR_) and a low-*T* activation energy linked to each other via *E*_a, low_ = (β – 1)*E*_a, high_, whereby *E*_a, low_ is affected by motional correlation effects.^25^ For 1/τ_c_, Arrhenius behavior is assumed, 1/τ_c_ = 1/τ_c, 0_ exp(−*E*_a, NMR_/(*k*_B_*T*)), where *k*_B_ denotes Boltzmann’s constant. β values smaller than 2 might also be interpreted in terms of a distribution of relaxation times. Such an analysis is, however, beyond the scope of the present study.

**Table 1:** Activation Energies *E*_a__, NMR_ (=*E*_a__, high_) as Derived from the BPP-type Fits of the Motion-Induced NMR Spin–Lattice Relaxation Rate Peaks Seen for the Nuclei ^7^Li and ^31^P^a^

	^7^Li^d^			^31^P			
peak	*E*_a, NMR_ (eV)	1/τ_c, 0_ (s^–1^)	β	*E*_a, NMR_ (eV)	1/τ_c,0_	β	type of motion
A	0.20 (0.18)	1.2 × 10^12^ s^–1^	1.92	0.18	2.9 × 10^11^ s^–1^	2	translational: fast Li^+^ intracage dynamics^b^
B	“0.26”	≈3 × 10^12^ s^–1^	≈1.7	0.44 (0.29)	≈2 × 10^11^ s^–1^	1.65	translational: Li^+^ exchange between the cages^c^
C				0.20 (0.15)	1.8 × 10^13^ s^–1^	1.74	rotational jumps of the polyanions^b^

a Values in brackets show the activation energies that do only refer to the low-*T* flank of the peaks. The prefactor 1/τ_c, 0_ and the asymmetry parameters β are also listed. β = 2 indicates symmetric peaks; in general, it takes values ranging from 1 to 2.

b Data taken from refs (22) and (29).

c This work.

d Underestimated value as the spin-lock rates are influenced by 1/*T*_1_.

Coming back to the spin-lock ^31^P NMR peak of Li_6_PS_5_I (peak B in Figure 2b), two important properties help us to identify its origin. First, the corresponding activation energy *E*_a, NMR_ of 0.44(4) eV, corresponding to the high-*T* flank (ω_1_τ_c_ ≪ 1), perfectly agrees with that obtained by dc conductivity spectroscopy (*E*_σ_ = 0.47 eV). It is, as probed by dc conductivity, sensitive to long-range ion dynamics because many jumps are probed during one Larmor precession. Note that the periodic time of one spin precession is defined by the angular locking frequency ω_1_, that is, in the high temperature limit of a given spin-lock NMR peak, we are dealing with the situation where 1/τ_c_ ≫ ω_1_ (= 20 kHz × 2π = 1.25 × 10^5^ s^–1^). Because of the perfect agreement seen in activation energies (*E*_a, NMR_ = *E*_σ_), we interpret the ^31^P spin-lock 1/*T*_1ρ_ NMR peak (labeled B in Figure 2b) as that directly mirroring the slow Li^+^ exchange processes between the Li-rich cages in poorly conducting Li_6_PS_5_I.

Further evidence of this assignment is provided by quantitatively comparing the diffusion coefficients obtained from dc conductivity measurements and the ^31^P spin-lock NMR data. If we use the Einstein–Smoluchowski equation, *D*_NMR_ = *a*^2^/(6τ_NMR_),^5,27^ to convert 1/τ_NMR_ estimated from the peak maximum appearing at 366 K into a diffusion coefficient, we obtain *D*_NMR_ = 4.56 × 10^–15^ m^2^ s^–1^. For *a,* we inserted the Li–Li distance between the Li-rich cages (*a* = 3.3 Å). *D*_NMR_ corresponds, according to the Nernst–Einstein equation, to an ionic conductivity of 5.4 × 10^–6^ S cm^–1^. Note that the Nernst–Einstein equation is, strictly speaking, only valid for diluted, non-interacting species; here, it serves as a tool to estimate the ionic conductivity. For comparison, the independently measured dc conductivity at 373 K is 5 × 10^–6^ S cm^–1^; at 353 K, it turned out to be 2 × 10^–6^ S cm^–1^.^22,33^ These values are in very good agreement with the one probed by diffusion-induced 1/*T*_1ρ_^31^P NMR spin–lattice relaxation.

In conclusion, while ^7^Li NMR is sensitive to the fast intercage ion dynamics, which are also indirectly seen by the ^31^P probes, with the help of spin-lock ^31^P NMR, it is possible to selectively study a much slower translational Li^+^ hopping process. We attribute this process to the much less frequent jumps between the Li-rich cages in crystalline Li_6_PS_5_I. Table 1 briefly summarizes our findings from both ^7^Li and ^31^P NMR.

Quite recently, we have seen that the introduction of the structural site disorder, by means of high-energy ball milling, leads to an increase of the direct current ion conductivity σ_dc_ of Li_6_PS_5_I by 2 orders of magnitude.^31^ This effect has not only been seen by dc conductivity measurements but is also present in electric modulus spectroscopy. Here, the position of the corresponding spin-lock ^31^P NMR 1/*T*_1ρ_ rates of this sample, which has been milled for 120 min in a planetary mill, are shown in the Arrhenius plot of Figure 2d as a dashed line. In agreement with the enhancement of long-range Li^+^ ion transport, we also recognize that the ^31^P NMR spin-lock peak, which is mirroring intercage ion dynamics being crucial for long-range ion transport, slightly shifts toward lower temperatures (347 K). However, this slight shift cannot fully explain the increase seen in σ_dc_ by factor of 100. As it has been revealed by ^7^Li spin-lock NMR, additional Li^+^*inter*cage processes, not necessarily seen by ^31^P NMR, became activated in disordered nano-Li_6_PS_5_I.^31^ As shown in the literature, the ^7^Li NMR 1/*T*_1ρ_ rates of the nanostructured sample pass through a broad peak located at ca. 265 K,^31^ which might reflect the source of rapid Li^+^ movements explaining the increase seen in nano-Li_6_PS_5_I.

Apart from this possible explanation provided by ^7^Li NMR, the current spin-lock ^31^P NMR peak of nano-Li_6_PS_5_I indicated in Figure 2d is to be characterized by an activation energy of 0.32 eV, which is by 0.12 eV lower than that of unmilled Li_6_PS_5_I. The same decrease in activation energy is also seen on the low-*T* flanks of the two corresponding peaks (Figure 2d); for unmilled Li_6_PS_5_I, the low-*T* flank is given by a slope resulting in 0.29 eV also see Table 1), while for nano-Li_6_PS_5_I, a value of only 0.18 eV is found, see Figure 2d. This comparison points to a flattening of the energy landscape in nanocrystalline Li_6_PS_5_I. At the same time it also indicates a larger distribution of relaxation times in materials with disordered structure as it is the case for nano-Li_6_PS_5_I. ^7^Li and ^31^P NMR data on nanocrystalline Li_6_PS_5_Cl and Li_6_PS_5_Cl are not yet available. As these compounds, particularly the Br one, are to be characterized by extensive site disorder^12^ resulting in very high conductivities, we do not expect that ball milling has such a strong effect on overall ion transport as it is seen for the poorly conducting and site-ordered Li_6_PS_5_I compound.

## 4 Conclusions

The combination of ^7^Li and ^31^P NMR spin–lattice relaxation measurements helped us in revealing two quite different motional processes in crystalline Li_6_PS_5_I. Whereas the ultrafast Li intracage diffusion process is best seen by ^7^Li NMR, the much less frequent hopping processes connecting the Li cages is revealed by ^31^P spin-lock NMR spin–lattice relaxation measurements carried out in the so-called rotating frame of reference. This method allows the detection of motional processes with correlation rates in the kHz regime. Here, the ^31^P nuclei do indirectly sense the Li^+^ exchange processes that are responsible for long-range ion transport in Li_6_PS_5_I. The activation energy associated with this process turned out to be 0.44 eV. It was extracted from a high-temperature flank of the 1/*T*_1ρ_(1/*T*) ^31^P spin-lock NMR rate peak. This value is in excellent agreement with that probed by macroscopic dc conductivity measurements (0.46 eV). Moreover, the self-diffusion coefficient determined by NMR at 366 K (*D*_NMR_ = 4.56 × 10^–15^ m^2^ s^–1^) does also agree very well with that dc conductivity spectroscopy is pointing at. Hence, ^31^P NMR turned out to be a complementary instrument, working at atomic scale, to fully characterize Li^+^ ion dynamics in thiophosphates. Results from complementary methods being sensitive to ion dynamics on different length scales are needed to entirely understand the complex dynamic processes in solid electrolytes. Considering the large family of lithium-ion conducting thiophosphates, we expect that ^31^P NMR will be highly useful to identify and characterize even more diffusion processes in this class of materials.
